# Transforming the Future Healthcare Workforce across Europe through Improvement Science Training: A Qualitative Approach

**DOI:** 10.3390/ijerph18031298

**Published:** 2021-02-01

**Authors:** Maria Cristina Sierras-Davo, Manuel Lillo-Crespo, Patricia Verdu, Aimilia Karapostoli

**Affiliations:** 1 Faculty of Health Sciences, University of Alicante, 03690 Alicante, Spain; manuel.lillo@ua.es; 2 Polytechnic School (EPSA), University of Alicante, 03690 Alicante, Spain; patriciaverdu91@gmail.com; 3 Department of Nursing, University of Peloponnese, 22100 Tripoli, Greece; aimilia219998@outlook.com

**Keywords:** Europe, thinking, improvement science, nursing students, qualitative research

## Abstract

Healthcare improvement science (HIS) is the generation of knowledge to cultivate change towards improving health systems performance. Our purpose was to evaluate the experience of European nursing students after an intensive one-week summer program conducted in 2019 at the University of Alicante in Spain. The educational intervention combined theoretical and practical HIS contents, with students from different countries, educational programs, and health systems. The intervention was evaluated under a qualitative approach through the open discussion group technique based on the method of participatory action research (PAR), with a total of 25 students who reflected about their experiences and perceptions during the intervention. The responses were used to improve the program’s contents, its didactics, and organization. Nursing empowerment, professional recognition, and healthcare research were some of the seven main categories identified through the systematic content analysis method triangulated by three experienced researchers. According to the students’ replies, values like compassion, respect, or empathy were identified as key elements of care. Promoting international students’ networking emerged as the key to creating a positive provision for change and the generation of improvement initiatives. Building a HIS culture may potentially provide future healthcare professionals with critical thinking skills and the resources needed to improve their future work settings.

## 1. Introduction

Over the period 1999–2010 the Bologna Reform in the European Union highlighted the importance of value-centered education across Europe in the field of health studies. In line with this, patient safety should be of upmost importance for healthcare professionals, while fundamental values like compassion, integrity, or human dignity, among others, are key to delivering the highest level of quality of care. However, those values are still not widely included in the training process of healthcare professionals in Europe and are not observed as part of improvement initiatives in the educational and healthcare fields [1,2,3,4,5,6].

From 2013 to 2015 the Improvement Science Training for European Healthcare Workers (ISTEW) project funded by the European Commission evidenced the gap in the provision of accredited health improvement science (HIS) education across Europe and outlined the need to improve quality of care services and related education. The most representative ISTEW outcomes were (a) the European HIS consensus definition, known as the Bled definition, (b) four HIS training modules, and (c) the Healthcare Improvement Science Evaluation Framework (HISEF) [7,8,9]. The Bled definition defines HIS in the European context as “the generation of knowledge to cultivate change and deliver person-centered care that is safe, effective, efficient, equitable and timely. It improves patient outcomes, health system performance, and population health” [9]. However, HIS status and understanding in other non-European countries such as the United States (U.S.) remain different. Since the 1980s improvement science has been developed extensively, focusing on health outcomes from an economic and efficiency perspective. In the U.S., the Institute of Health Improvement (IHI) has been focused for decades on the creation of specific improvement education, its implementation in healthcare contexts, and dissemination in their healthcare system [8]. Across the European countries, differences among HIS understanding and practice have been evidenced. In fact, a higher level of development is observed in the English-speaking countries such as the United Kingdom and Ireland. In the European educational field specifically, the differences are even more evident. For instance, in Slovenia only 4% of the European Quality Assurance Register for Higher Education (EQUAR) courses include HIS contents, a figure similar to that of Italy (7%), followed by Poland (10%), and far behind England (27%) and Romania (25%) [8].

As stated before, based on the previous gap analysis of nursing studies in Europe conducted during the ISTEW Project there is a lack of specific training for nurses focused on the following items: development of improvement-based and critical thinking, quality improvement measurements, systems thinking, and safety practices [8]. Therefore, those items were the ones upon which the ISTEW modules and the contents of the Alicante Summer Program were based. The University of Alicante in Spain, as a partner team, promoted HIS culture and prospectively used the ISTEW outcomes by organizing an Annual International Summer Program. The “Immersion in HIS” course started in July 2016 and was repeated yearly until 2019 [7,8]. Participants were nursing students from different European universities (Scotland, Ireland, Finland, and Spain), and were therefore from different cultures, with distinct types of health system organization and professional competencies. Such international education led to a discussion on how value-centered healthcare education focusing on HIS should be considered, while analyzing the differences and similarities amongst cultures. Students had the chance during the training to propose improvement initiatives in their own real contexts and discuss what other colleagues from other cultures were doing [1]. Along the four Summer Programs, the HIS Evaluation Framework (HISEF) created throughout the ISTEW project was used as the evaluation tool which included participants’ qualitative and quantitative data through different questionnaires based on Kirkpatrick’s Learning Evaluation Model [10,11,12]. To support the data collected through the evaluation framework, new dynamics were introduced in 2019. The research presented focuses on this new section where qualitative data were collected after exploring the experience and perception of European nursing students regarding HIS after an intensive one-week summer program.

## 2. Materials and Methods

### 2.1. Educational Intervention, Qualitative Method, and Techniques

A practical and theoretical educational intervention regarding HIS was conducted consecutively from 2016 to 2019, focusing on the four main HIS modules developed by the ISTEW project: (a) the development of improvement-based and critical thinking, (b) quality improvement measurements, (c) systems thinking, and (d) safety practices. For our research purpose we concentrated on the qualitative data collected in the 2019 course. The educational intervention was evaluated under the scope of participatory action research (PAR), which was selected as the qualitative method. Within PAR, the subject becomes the protagonist and participates in the change itself. Citing Cassell and Symon [13], PAR enables participants to confront their experiences and existing conflicts with others, particularly in healthcare provision to the patients. The transition from object of study to subject protagonist is carried out by cyclic processes of reflection–action–reflection where the researcher continuously evaluates each intervention, interacting constantly with the target study population [14]. We understood that the inclusion of all the course users and all the educators participating in this intervention would determine the success of the implementation of HIS knowledge in the future healthcare workforce [15].

Although we used the HISEF as the evaluation tool of the HIS learning, which included open-ended and closed questions together with Likert scales, we considered this insufficient for our qualitative goal. For that reason, further qualitative research to capture students’ personal perspectives and experiences was needed. In order to fill this gap, a plenary discussion and brainstorming session was conducted at the end of the intervention in 2019, providing an approach to the participants’ perceptions and experiences. The session had four main topics: (a) take-home ideas, (b) values learnt, (c) previous HIS experience, and (d) initiatives that students would implement to improve their local settings and also the recently visited ones during the Summer Program.

### 2.2. Setting and Procedures

The educational intervention for healthcare future professionals and its evaluation was conducted in July 2019 at the University of Alicante. Since the ISTEW project ended, this course has been the only implementation initiative with regard to the specific educational modules created in the project. It consisted of a one-week 50-h program divided into theory and practice. Students had the chance to visit Spanish public and private hospitals as well as primary health care centers, observing, detecting, and discussing similarities and differences with regard to their healthcare contexts and contrasting such practical experience with the knowledge achieved in the theoretical sessions. The purpose of this intervention was to develop their theoretical and practical knowledge about HIS contents and values, promoting critical thinking, developing improvement-based thinking and behavior, creating awareness, and consequently generating a HIS culture. During the course, students created their own projects designing HIS interventions in practice by using scientific HIS evidence and sources (e.g., indicators, questionnaires, interviews etc.) and presented their ideas in a dynamic environment where all students could make their input and interact to one another. For our research purpose we conducted the discussion session in the main classroom used for the course at the University of Alicante on the last day once the program had been fully completed.

### 2.3. Participants

Twenty-five nursing students from other European Higher Education Institutions such as the University of The West of Scotland in the United Kingdom, the Waterford Institute of Technology in Ireland, the Laurea University of Applied Sciences in Finland, and the University of Alicante itself participated. All of them agreed to be part of the plenary discussion and participated in the cyclic process of reflection–action–reflection based on PAR principles in which the researchers evaluated continuously each intervention, interacting constantly with them [16].

### 2.4. Data Collection and Analysis

Students’ experiences through the course were collected from the discussion session conducted. Notes were taken manually by one researcher. Another experienced researcher moderated the session in which students and educators participated, and the other researcher was the observer. The full transcribed notes are in the Supplementary Material (Document S1). The data content analysis was the method of analysis chosen and was carried out throughout a triangulation process in which three experienced qualitative researchers participated. Content analysis is a systematic analysis method that makes inferences in this case from the participants’ experiences expressed in the open session and observed by the researchers. The results were classified firstly following the four main topics that guided the discussion: Take-home ideas, values learnt, previous HIS experience, and initiatives that students would implement to improve their local settings and also the recently visited ones during the Summer Program. The three researchers participating in the analysis decided to classify the answers to the first three topics into categories according to the number of times repeated, while the results of the fourth topic were gathered by country, since the analysis of the data showed that the content of the students’ answers was associated with their place of origin.

## 3. Results

About the first topic, researchers explored the main idea that students referred as having learnt. After the analysis of this topic, eight categories came up corresponding to the most-repeated ideas (Table 1).

Continuing with topic 1, in Table 2 eight categories have been gathered according to whom is responsible for them: “Internal” indicates that it is the student/future professional who is responsible for the action and “External” refers to when the responsibility lies with another person/organization.

In the second topic the most significant value learnt for each student was highlighted. After the analysis of the answers, classification was performed with regard to the five most repeated values for the students. In order, the most repeated value was Teamwork, followed by Respect, Passion, and Humanization of Care/Compassion, with Communication being the least repeated.

Thirdly, the question “What would you improve in this context and in your context” was asked. This section is about the exchange of improvement, which reflects the different improvements and/or changes that students think can be made both in Spain, where the course took place, and in their country of origin. For the response analysis, the thematic units extracted from the first question have been reused, defined, and finally a selection of the most repeated answers has been presented in Table 3.

Finally, in the Table 4 the fourth topic discussed, “Have you ever had any type of improvement science subject or previous experience?”, was analyzed per country and the responses were grouped after reaching a consensus among the participants themselves.

## 4. Discussion

This study aimed to evaluate the European nursing students’ experiences and perceptions after an educational intervention on healthcare improvement science (HIS). This qualitative study and others have demonstrated how relevant healthcare improvement science is at all professional and educational stages for the nursing profession [1,8,10]. Developing and evaluating this educational intervention from the perspective of the ISTEW project modules will contribute to the ISTEW project main aim by taking a step towards standardizing HIS culture across Europe [7,9]. During the implementation of the modules, the researchers’ team agreed to evaluate the intervention every year and integrate students’ feedback and needs through participatory action research methodology according to the experience presented in this manuscript. The inclusion of the open session discussion in 2019 permitted a deeper exploration of students’ feedback. The study team understood how important it is to have a full understanding of the student’s perspective to build bridges between theory and practice, enabling them to succeed in this transition process.

This research contributes to an understanding of how healthcare improvement science education provides nursing students with the confidence to make changes in their future work settings, delivering safe, effective, person-centered, efficient, equitable, and timely care [9]. To assure and follow up on the lessons learned as well as implementation in the work settings by students, further prospective research is needed [17,18]. Future courses with the new HISEF version combined with qualitative PAR are being planned with a virtual format due to the SARS-CoV-2 (COVID-19) pandemic.

The methodology used is effective in capturing student transformation, experiences, and perceptions during the course. The new section during the 2019 course and presented in the tables was perfectly combined with the HISEF to deeply understand students’ perspectives and experiences. In relation to the main categories and topics identified, a tendency can be observed. In accordance with the results obtained in the literature reviewed conducted by Lillo et al. [8], keywords like “nursing empowerment”, “nursing research” or “healthcare systems” are important with regard to student involvement with HIS education. However, as also mentioned in the previous study, the disparities among European countries create difficulties in healthcare improvement science standardization. This context can be seen as a weakness, but the authors used it as a strength to increase knowledge exchange among students due interactions during the course. From this research and previous publications on the field a conclusion can be made: Due to HIS disparities, educational interventions should include an international perspective. It has been observed that in Europe, HIS is understood and practiced in different ways according to the country. If a more comprehensive and broader perspective on HIS is to be achieved, educators, students, and finally healthcare systems should benefit from international educational exchanges and networking [8]. There is evidence suggesting that supporting staff at the early stages is the key step to driving systems into sustainable changes to promote patient-centeredness [19]. On this basis, the improvement of science education early in nursing careers relies on a common understanding of best practices and improvement methods that have the potential to redirect healthcare settings towards values such as safety or compassion, with a natural impact on patients’ quality of care [20]. Improvement science has the potential to develop, but all related interventions must be evaluated [21]. HIS benefits need to be evidenced and all efforts in its development will be crucial for the future of healthcare systems [22,23].

### Limitations

The content analysis method selected had a potential risk regarding the researchers’ implication when analyzing the data and drawing conclusions. To prevent this, three researchers participated in the analysis process through an analysis triangulation. On the other hand, quantitative data obtained from the HISEF should be prospectively compared with the qualitative information collected, improving both evaluation methods in order to capture the students’ experiences as accurately as possible. Moreover, students from other countries and from other health professions should be included towards to provide more evidence. However, despite the limitations, this paper is a starting point that provides useful information about nursing students’ interactions within a global HIS perspective.

In relation to the qualitative technique used, the type of open discussion group ran the risk of leaving out feedback from those participants who were less self-confident in expressing their opinion in public. In order to avoid this, all participants were asked one-by-one in a safe and open atmosphere, encouraging them to express their opinions and facilitating the discussion among all members. Finally, further evaluation rounds would be needed in future educational interventions in order to see if the last HISEF version after the 2019 course better captured quantitative data and whether the results were coherent with the qualitative data collected through the open discussions. Further course editions are planned as soon as face-to-face education and travel between countries without restrictions are possible.

## 5. Conclusions

The new summer course evaluation process was conducted successfully, and the students’ experiences and perceptions were well captured, as detailed previously. Students improved their critical thinking and knowledge in HIS and professional values and learned about the ways things are done in other cultural contexts. The educators also had the chance to improve the didactics, contents, and organization of the course. The PAR method is useful for students to reflect about course contents and ideas for improvement. An increase in students’ motivation, inspiration, and willingness for a transformation based on improvement emerged. Nevertheless, with the current available data long-term consequences in healthcare systems cannot be demonstrated at this early stage. A longer follow-up phase for students is needed.

## Figures and Tables

**Table 1:** Categories regarding the students’ main ideas on healthcare improvement science (HIS).

Categories	(Times Repeated) Percentage
Nursing empowerment	(10) 11.36%
Healthcare flat system/organization	(26) 29.54%
Healthcare professionals’ motivation	(6) 6.81%
Nursing research	(7) 7.95%
Job appreciation and recognition	(18) 20.45%
Values in healthcare	(12) 13.63%
Communication between team members	(5) 5.68%
Professional development	(4) 4.54%

**Table 2:** Category classification per responsible agent.

Internal	External
Empowerment	Flat system
Motivation	Communication
Recognition (of oneself)	Recognition (of others)
Values (of oneself as a person)	Values (of the company/system)
Research	Professional development

**Table 3:** Topics and student quotations.

Topic	Student’s Quotations
Nursing empowerment	“Better understanding of empowerment in nursing.” (C1) “We don’t have many male nurses there, we feel more empowered now to inspire others.” (C2) “We need more empowerment. We are consumed by the system working a lot but we don’t think about doing something further” (C3)
Communication between team members	“Teamwork. In Finland they sometimes don’t even talk to each other.” (C4) “Collaboration between different professionals.” (C5) “Collaboration between other health professionals is impossible. I see this here.” (C6)
Healthcare professionals’ motivation	“I have gained in motivation, inspiration and improvement. We have a lot of motivation now. The course has inspired us.” (C7) “We have seen a lot of motivation among nurses in Spain” (C2) “The Spanish nurses are very positive and nice.” (C8)
Healthcare flat system/organization	“I like the concept of healthcare flat system but I don’t see it in reality.” (C9) “We don’t have key people in key positions.” (C3) “In Finland our system is more rigid.” (C10)
Job appreciation and recognition	“Nurses in Spain are highly respected.” (C11) “We nurses should feel more proud, not say more: I’m just a nurse.” (C12) “There should be more recognition if you keep studying, it should translate into more salary.” (C13)
Nursing research	“Now we feel the need to do research.” (C1) “We understand that further research is needed.” (C14) “This course opened my mind about research.” (C15)
Professional development	“In Greece, nurses that do research are increasing.” (C6) “More training and updating is needed.” (C7) “Recognition of the visible effort in increasing wages is needed.” (C16)
Values in healthcare	“Positive about life and work.” (C8) “Family involvement.” (C17) “More respect and humanization of care.“ (C14)

**Table 4:** HIS experiences per country.

Country	Answer
Greece	“Two modules of management but is more about organizations not improvement or empowerment. We get more empowerment from the community nurse. We have some research subjects. I think the training is improving, the most important is that our teachers are nursing leaders. In other subjects the teachers are MDs, they don’t even recognize them. We don’t have specific laws that protect us.” (C17)
Finland	“We have management but not anything similar to this. There are some improvement courses but are not always in all universities or accessible to everyone. It’s something more about our university, not a country standard. In Laurea we have a subject including improvement but not sure about others.” (C18)
Scotland	“We had before research at university but improvement is more in hospital not at university or not in my case. However, the promotion of research as carried out in this course is not as strong.” (C19)
Spain	“Here the nurse works on many initiatives to improve specific aspects of health care but HIS is not recognized as a concept or discipline. Except for this course we are not aware of any further specific training.” (C20)

## Data Availability

The data is presented in the paper.

## Supplementary Materials

The following are available online at https://www.mdpi.com/1660-4601/18/3/1298/s1, Document S1: The full transcribed notes.
