# Supplementary material for: Transforming the Future Healthcare Workforce across Europe through Improvement Science Training: A Qualitative Approach
Source: Int J Environ Res Public Health. 2021 Feb 1;18(3):1298. doi: 10.3390/ijerph18031298 (PMC7908231; doi:10.3390/ijerph18031298)
Supplement: Supplementary file 1 [file ijerph-18-01298-s001.pdf]

Document S1. The full transcribed notes.

The four questions conducted during the group discussion are:

1. What new ideas and concepts have you learned?
2. What would you improve in this context (Spain) and in your own?
3. What values have you learned in relation to care?
4. Have you ever had contact with HIS? Please give us a brief summary of the basic aspects of your experience.

The answers to questions 1 and 2 have been classified into 8 categories. The letters assigned at the beginning can be seen in the corresponding answers:

- **(A)** Nursing Empowerment. 21.43%
- **(B)** Communication between team members 7.14%
- **(C)** Healthcare professionals' motivation 14.29%
- **(D)** Healthcare flat system/ organization 16.67%
- **(E)** Job appreciation and recognition 11.90%
- **(F)** The relevance of doing research and 14.29%
- **(G)** Educational opportunities/ career development 2.38%
- **(H)** Values in healthcare 11.90%

1. What new ideas and concepts have you learned?

1. **C1** Bigger picture of what is nursing empowerment/students empow **(A).** Pressure to do research **(F).** Before they only think about work now she feels she should also research. Reducative pages of an article. Scientific literature should be easy to read. She is thinking about general practice.
2. **C7** Motivation, inspiration and change things. We have a lot of motivation. The course has inspired us **(C).**
3. **C9** I like the universal free healthcare system but with our Finnish resources. I liked the system **(D).**
4. **C4** Communication between the team **(B).** In Finland sometimes they don't talk to each other.
5. Family as a value **(H).** How culture and family are integrated in the Spanish system. Through the nurses work. Also the motivation in Spanish nurses is higher **(C).**
6. **C8** Positive mind about life and work **(H).** Spanish nurses are really positives, smiler and nice **(C).**
7. Same as 5. Nursing appreciation as a profession **(E).**
8. This course should be a subject at schools. Nurses in clinical work they don't understand what are doing. This is about nursing empowerment **(A).** Being in the centre. In Finland every nurse with a Master is in a too high level then the areas working don't see this an example.
9. **C15** This course opened my mind about research **(F).** I've never thought about them in my 10 years practice. Now, I'm starting to think if I should do something else.
10. **C5** The course helps me again a fresh mind. I worked in research **(F)** before but never link to nursing. Now I feel about nursing empowerment **(A).** I have learnt about flat organizations **(D).** Collaboration between different professionals **(B).**
11. **C2** Inspiring others around me **(C).** We don't have male nurses there, we feel more empowered now to inspire others **(A).** We can improve the quality of care **(H)** if we inspire others. Nursing prescribing Spanish model liked and inspired us a lot **(C).**
12. In Spain nurses are more appreciated **(E)** than in Finland. Is a point of social identity and social recognition.
13. Flat organizations **(D)** a one ide liked me. Reducing the barrier of gauder in nursing.
14. Appreciation **(E)**, empowering nurses as ideas **(A).**
15. Spanish healthcare system **(D)** liked me.
16. Passion about nursing **(C)**, the respect people **(H)** have for you here is not happening in Finland.
17. Idea to be curious about what surrounds me. Develop myself further **(A).**
18. **C11** Nurses in Spain are well respected **(E).** This respect should be also in Scotland. (Scottish Student)
19. The course has his listened how important is research in our daily job **(F).** (Scottish Student)
20. **C3** Grece. Possibilities that nurses have here **(D).** In our country there is no many master options or masters **(A)** /nurses are not as appreciated as here **(E).** They called us "sisters" not even by our name. Empowerment **(A).** We need to be more empower. We are consumed by the system working a lot but we don't think about doing something further. We don't have key people in key positions **(D).**

21. C6 Research **(F)**. In Greece nurses that do research are increasing **(G)**. Collaboration between other health professionals **(B)** is impossible. I see this here. (Greece)
22. Research **(F)**. Participating in projects, explore more.
23. Flat organizations **(D)**. How I have a new perspective of what it is nursing.
24. Critical thinking **(H)**. The course focused me to think more.
25. I agree with the rest. For me I liked the most empowerment **(A)**.

2. What would you improve in this context (Spain) and in your own?

1. Family treatment home **(H)**. To take here not sure.
2. Home: nurses can be more proud and satisfied **(E)**. To take here not sure
3. System there. Here the recognition **(E)** of higher education to create band or salary recognition.
4. There: proud of our work **(E)**. Here: not sure.
5. C7 There: more training and being updated **(G)**. Here: more improvement **(H)** on the field and better implementation **(D)**.
6. C10 There: The child wards here are really friendly **(D)**. Here: More strict aseptic policy **(D)** in Finland we are more strict.
7. There: appreciation of nurses **(E)**. Here: More strict aseptic policy in Finland we are more strict.
8. There and here: nursing empowerment **(A)** and magnet hospitals mindset **(D)**
9. C12 There and here: nurses to be more proud not say more "I'm just a nurse" **(E)**
10. C13 Here: better recognition in salary and promotion if you continue studying **(E)**. There: to do more improvement **(H)**.
11. There: direct communication **(B)**. Float organization **(D)**. Be brave and say what you think. Here: More strict aseptic policy in Finland we are more strict **(D)**.
12. There: proud to be a nurse **(E)**.
13. There: flat organization. Here: family not having so much pressure more home care and resources **(D)**.
14. There: appreciation **(E)** better, salaries. Here: better salaries **(D)**.
15. There: free healthcare system **(D)**. Here: not sure.
16. There: communication in the team doctors and nurses **(B)**. Here: not sure.
17. There: proud of nurses **(E)**. Here: More strict aseptic policy in Finland we are more strict **(D)**.
18. C17 Scotland There: family involvement **(H)**. Here: More strict aseptic policy in Finland we are more strict **(D)**. Using move gloves **(D)**.
19. Scotland: There: friendly pediatric areas **(D)**. Here: recognition of educational level **(E)**.
20. Greek: There: flat system **(D)** + appreciation **(E)**. Here: improve the shifts **(D)**.
21. C14 Greek: There: more respect **(H)**. Here: keep going on research/education **(F)**.
22. C16There: protocol for children at ER not waiting so much **(D)**. Here: flexible system **(D)**, more opportunities **(G)** and recognition **(E)** of the effort on salaries and work career development **(G)**.
23. There: learn to include family **(H)**. Here: improve **(H)** elderly care.
24. There: timetable, improve the shifts **(D)**. Here: N/A
25. There: flat organization **(D)** appreciation of nursing **(E)**. Here: NIA

3. What values have you learned in relation to care?

1. Kindness
2. Understanding
3. Open mind
4. Respect
5. Appreciation
6. Passion
7. Sharing ideas - Collaboration
8. Compassion
9. Passion
10. Mindset
11. Excellence
12. Respect
13. Listening - Communication
14. Empathy
15. Team Work
16. Improvement
17. Communication

18. Commitment
19. Empowering
20. Progress
21. Team Work
22. Quality
23. Experience
24. Team Work
25. Compassion

4. Have you ever had contact with HIS? Please give us a brief summary of the basic aspects of your experience.

Country	Answer
Greece	"Previously we have had courses on hospital management, but they have been related to systems not improvements or HIS. The nurse in Greece feels more empowered in the community. Training in general is improving and more and more research topics are being studied, however the most important thing for us is that our leaders belong to the nursing field. Usually the teachers are doctors and they do not even recognize our discipline. We have no specific laws to protect and promote us." **C17**
Finland	"We have subjects in management but not anything like this. There are some courses in health improvement, but they are not always in all universities or accessible to everyone. In Finland, a lot depends on the universities. In Laurea we have a subject that includes HIS, but we don't know if it exists in other universities in the country." **C18**
Scotland	"In Scotland there are specific programs in HIS at universities and hospitals. However, the promotion of research as carried out in this course is not as strong." **C19**
Spain	"Here the nurse works on many initiatives to improve specific aspects of health care but HIS is not recognized as a concept or discipline. Except for this course we are not aware of any further specific training" **C20**
